# Application of 4-channel neuromuscular electrical stimulation in a chronic stroke patient with dysphagia: A case report

**DOI:** 10.1097/MD.0000000000048947

**Published:** 2026-05-22

**Authors:** Jee Hyun Suh, Jaewon Lee

**Affiliations:** a Department of Rehabilitation Medicine, Seoul National University Bundang Hospital, Seoul National University College of Medicine, Seongnam-si, Gyeonggi-do, South Korea.

**Keywords:** 4-channel NMES, chronic stroke, dysphagia

## Abstract

**Rationale:**

Dysphagia is a frequent complication after stroke, often resulting in aspiration, malnutrition, and diminished quality of life. Conventional swallowing rehabilitation, including 2-channel neuromuscular electrical stimulation (NMES), may have limited benefits in chronic cases. A newly developed 4-channel NMES delivers sequential stimulation to mimic physiological swallowing.

**Patient concerns:**

A 63-year-old man experienced persistent dysphagia 25 months after a left hemispheric infarction. Despite prolonged conventional swallowing rehabilitation combined with 2-channel NMES, he remained restricted to pureed foods (International Dysphagia Diet Standardisation Initiative level 4), required frequent suctioning, and showed severe aspiration on videofluoroscopic swallowing study (penetration-aspiration scale score up to 8).

**Diagnoses:**

The patient was diagnosed with chronic poststroke dysphagia with severe aspiration confirmed by videofluoroscopic swallowing study.

**Interventions:**

The patient underwent an 8-week course of 4-channel NMES applied twice daily in combination with conventional swallowing rehabilitation.

**Outcomes:**

Following the intervention, the patient’s diet advanced to International Dysphagia Diet Standardisation Initiative level 6, penetration-aspiration scale scores improved to 1 to 3, suctioning was no longer required, and both the Eating Assessment Tool-10 and Swallowing Quality of Life Questionnaire scores markedly improved.

**Lessons:**

This case suggests that 4-channel NMES may be a promising treatment option for chronic poststroke dysphagia refractory to conventional swallowing rehabilitation and warrants further validation in larger studies.

## 1. Introduction

Dysphagia represents a prevalent and clinically important sequela of ischemic stroke. According to a previous study, 43.7% of chronic stroke patients were found to have dysphagia.^[1]^ Dysphagia is associated with medical complications such as malnutrition, dehydration, and aspiration pneumonia, as well as psychosocial consequences including reduced enjoyment of eating, social withdrawal, and impaired quality of life.^[2]^ Dysphagia is associated with prolonged length of hospital stay, increased healthcare costs, and a higher likelihood of long-term institutionalization.^[3]^

Decreased laryngeal elevation due to pharyngeal muscle weakness is a major cause of dysphagia, leading to aspiration and pharyngeal residue during swallowing.^[4]^ 2-channel neuromuscular electrical stimulation (NMES) has been used to treat dysphagia, particularly in acute poststroke patients, under the assumption that it improves pharyngeal muscle weakness. Previous studies have shown that 2-channel NMES can improve the penetration-aspiration level, effectively increase the forward and upward movement of the hyoid bone, enhance quality of life, reduce the rate of complications, and improve swallowing function in patients with poststroke dysphagia.^[5,6]^ However, the effectiveness of 2-channel NMES alone remains unclear.^[7]^ Furthermore, recent structured studies have reported that 2-channel NMES did not accelerate swallowing recovery in the acute and subacute stages of poststroke dysphagia.^[8]^ In an effort to address the limitations of 2-channel NMES, a 4-channel NMES device was developed. Clinical studies have shown that this approach can reduce post-swallow pharyngeal residue, promote more effective swallowing, and activate a more physiologic trajectory of hyoid bone movement in patients with dysphagia^[9,10]^

However, most studies on NMES for dysphagia have focused on patients in the acute or subacute phase following stroke. As a result, there is limited evidence for its effectiveness in patients with chronic stroke, making treatment decisions in clinical practice challenging. This case report aims to present the therapeutic effects of 4-channel NMES in a patient with dysphagia more than 2 years poststroke.

## 2. Case presentation

### 2.1. Patient

**Figure 1.**
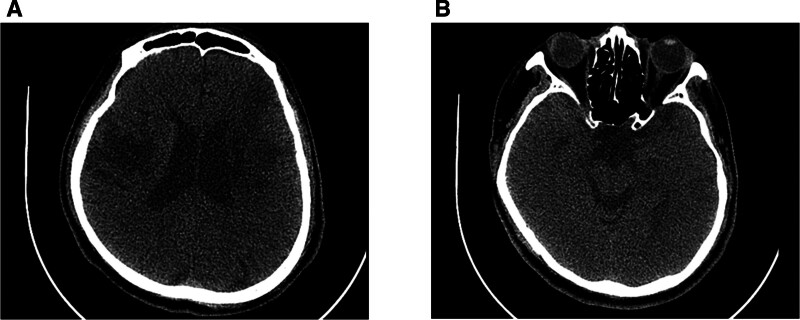
Brain CT images obtained 8 months after the second cerebral infarction. (A) Chronic infarct lesions are observed in both cerebral hemispheres; (B) No definite abnormal changes are noted in the brainstem region. CT = computed tomography.

**Figure 2.**
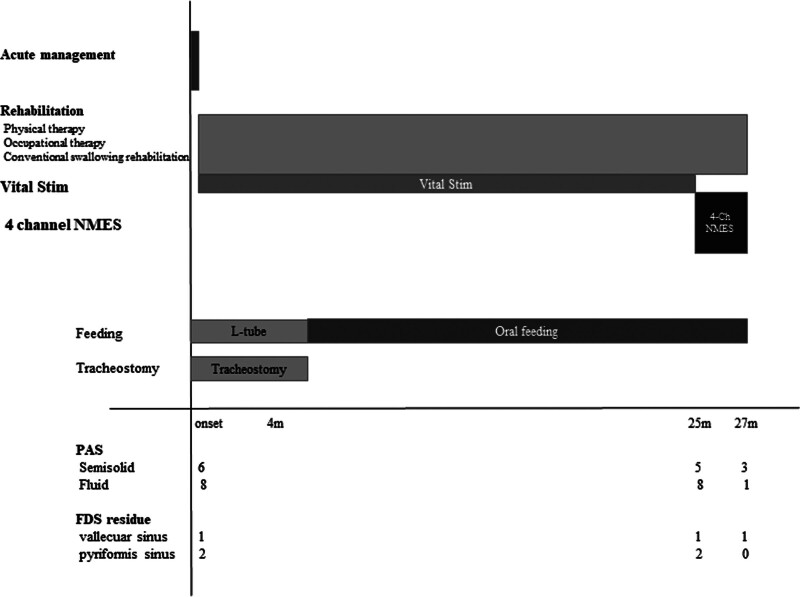
Summary of the patient’s clinical course, including rehabilitation progress and changes in swallowing function after the second cerebral infarction. FDS = Functional Dysphagia Scale, NMES = neuromuscular electrical stimulation, PAS = penetration-aspiration scale.

A 63-year-old male patient, a board-certified family medicine physician, experienced a right hemispheric infarction 12 years prior, from which he recovered without any residual deficit, including no swallowing impairment. Subsequently, 25 months ago, he suffered a left hemispheric infarction, which resulted in right hemiplegia, dysphagia, dysarthria, and right central facial palsy (Fig. 1). On videofluoroscopic swallowing study (VFSS) performed after onset, aspiration was observed with a Penetration-Aspiration Scale (PAS) score of 6 for semisolid and 8 for fluid, along with vallecular and pyriform sinus residue graded as Functional Dysphagia Scale (FDS) grade 1 and grade 2. At the time of stroke onset, he was receiving feeding via a Levin tube and had a tracheostomy.^[10,11]^ He underwent comprehensive rehabilitation therapy 1 week after onset, including 2-channel NMES using the VitalStim system (DJO Global, Vista), dysphagia therapy, physical therapy, and occupational therapy, during a 4-month admission to a tertiary university hospital’s department of rehabilitation medicine, followed by a 21-month stay at a specialized rehabilitation hospital. By 6 months post-onset, oral feeding at Functional Oral Intake Scale level 4 and tracheostomy sealing were achieved. However, his dysphagia remained stationary thereafter. He was able to consume only pureed foods consistent with grade 4 of the International Dysphagia Diet Standardization Initiative (IDDSI), and frequent suctioning was required. A VFSS performed at 25 months post-onset revealed persistent swallowing impairment, with a PAS score of 5 for semisolids and 8 for both thin and thick liquids. As a result, oral feeding with thickened liquids (IDDSI 4) was maintained (Table 1). The patient exhibited reduced tongue mobility, impaired bolus formation, and diminished mastication. Post-swallow residue was observed on the tongue surface. Weakness of the tongue base and inadequate laryngeal closure led to laryngeal penetration. Furthermore, insufficient pharyngeal pressure generation resulted in vallecular residue (Grade 1 on the FDS) and pyriform sinus residue (Grade 2 on the FDS), with overflow of the latter causing aspiration (Fig. 2). There was no evidence of delayed triggering of the swallowing reflex in this patient.

**Table 1:** Clinical course and dysphagia.

	Before 4-channel NMES	After 4-channel NMES
FOIS	4	6
Body weight (kg)	63.8	66.4
BMI (kg/m^2^)	19.69	20.49
NIHSS Item 4: Facial Palsy	3	1
NIHSS Item 10: Dysarthria	2	1
PAS (semisolid)	5	3
PAS (Fluid)	8	1
Vallecular retention	1	1
Pyriformis sinus retention	2	0
EAT-10	39	14
SWAL-QOL	54	137

BMI = body mass index, EAT-10 = Eating Assessment Tool-10, FOIS = Functional Oral Intake Scale, NIHSS = National Institutes of Health Stroke Scale, NMES = neuromuscular electrical stimulation, PAS = penetration-aspiration scale, SWAL-QOL = Swallowing Quality of Life Questionnaire.

The study was approved by the Institutional Review Board of Seoul National University Bundang Hospital (Institutional Review Board No. B-2506-979-701). Written informed consent was obtained from the patient for participation and publication of this case report, including the use of clinical data and images.

### 2.2. Intervention

**Figure 3.**
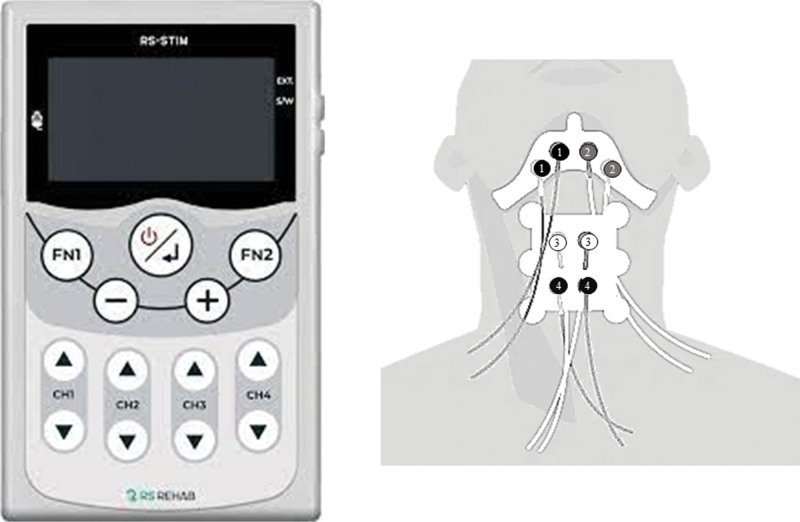
Configuration of the NMES device and electrode placement. (A) The 4-channel NMES device used in this study; (B) Electrode placement sites: Channels 1 and 2 were positioned just above the hyoid bone and behind the mandible, approximately 1 cm lateral to the midline on the right and left sides, respectively. Channel 3 was placed over the superior poles of the thyroid cartilage, and Channel 4 was positioned medial to the sternocleidomastoid muscle and inferior to the thyroid cartilage. Reproduced with permission from RS REHAB Co., Ltd. NMES = neuromuscular electrical stimulation.

An 8-week course of 4-channel NMES therapy for dysphagia was administered using the 4-channel NMES device (RS-STIM 1.0, RSRehab) (Fig. 3A). The electrode placement was guided by anatomical landmarks and confirmed through manual palpation (Fig. 3B). Electrodes for Channels 1 (right) and 2 (left) were positioned just above the hyoid bone and behind the mandible, approximately 1 cm lateral to the midline, to stimulate the digastric and mylohyoid muscles. Channel 3 electrodes were applied to both sides of the upper thyroid cartilage to activate the thyrohyoid muscles. Channel 4 electrodes were positioned medially to the sternocleidomastoid muscle and below the thyroid cartilage, targeting the sternohyoid, omohyoid, and sternothyroid muscles (Table 2).^[10]^ The stimulation parameters were adopted from a previously established protocol. The electrical pulses were delivered at a frequency of 80 Hz with a pulse duration of 300 microseconds and an interphase interval of 100 microseconds. Stimulation began with Channels 1 and 2, followed by Channel 3 after a delay of 150 milliseconds and Channel 4 after 250 milliseconds. The durations of stimulation for Channels 1, 2, 3, and 4 were 1200, 1200, 1050, and 950 milliseconds, respectively, with all channels terminating simultaneously in each stimulation cycle.^[10]^ He underwent 4-channel NMES twice daily for 30 minutes per session, 7 days a week. Adherence to NMES was verified through the patient’s daily treatment logs, which were used to monitor compliance with the prescribed sessions. The initial stimulation intensities were set at 3.5 mA, 3.5 mA, 3.0 mA, and 5.5 mA for channels 1 through 4, respectively. As his tolerance improved, the intensities were gradually increased to 4.5 mA, 4.5 mA, 4.0 mA, and 7.0 mA, respectively. During the 8-week period of 4-channel NMES treatment, conventional swallowing rehabilitation (consisting of oral and pharyngeal muscle strengthening exercises and compensatory strategies) was concurrently administered 5 times per week, 30 minutes per session.

**Table 2:** Electrode attachment sites for the 4-channel NMES.

Channel	Side	Placement location	Target muscles
1	Right	Just above the hyoid bone and behind the mandible, approximately 1 cm lateral to the midline	Digastric, Mylohyoid
2	Left	Same as Channel 1, mirrored on the left side	Digastric, Mylohyoid
3	Midline	Over the superior poles of the thyroid cartilage	Thyrohyoid
4	Midline	Medial to the sternocleidomastoid muscle and below the thyroid cartilage	Sternohyoid, Omohyoid, Sternothyroid

NMES = neuromuscular electrical stimulation.

### 2.3. Results

Beginning in the third week of treatment, the patient reported a subjective improvement in swallowing ease, and by the fourth week, suctioning was no longer required. Prior to stroke onset, his body mass index was 22.22 kg/m^2^; however, by 25 months post-onset, it had decreased to 19.69 kg/m^2^. Following 8 weeks of 4-channel NMES treatment, his body mass index increased to 20.49 kg/m^2^. Dietary intake improved from IDDSI level 4 (pureed) to level 6 (soft and bite-sized), and Functional Oral Intake Scale improved from level 4 to level 6. For liquids, intake progressed from IDDSI level 4 (extremely thick) to level 2 (mildly thick). The patient’s Eating Assessment Tool-10 score improved from 39 to 14, and the Swallowing Quality of Life Questionnaire score increased from 54 to 137. Improvements were also observed in central facial palsy and dysarthria. After 8 weeks of 4-channel NMES, follow-up VFSS showed improvement, with a PAS score of 3 for semisolid, and 1 for both thin and thick fluids. Pyriform sinus retention was also improved, with no significant residue observed (Table 1). Clinically, the patient initially had difficulty with lip opening/closing, tongue protrusion, and lateralization, but showed noticeable improvement following the 8 weeks of 4-channel NMES intervention. No adverse events, including skin irritation, discomfort, or cardiovascular changes, were observed during NMES sessions.

## 3. Discussion

This case demonstrates that a newly developed 4-channel NMES may serve as an effective therapeutic option for dysphagia unresponsive to conventional swallowing rehabilitation and 2-channel NMES in a patient with chronic stroke persisting for over 2 years. The 4-channel NMES was shown to reduce pharyngeal residue, improve the PAS score, and consequently enhance the patient’s quality of life. In addition, improvements were also observed in tongue movement and facial muscle function.

Dysphagia in chronic stroke patients is often less responsive to treatment than in acute stroke patients due to factors such as reduced neuroplasticity, chronic tissue changes, and persistent neuromuscular impairments.^[12–14]^ Additionally, the initial severity of dysphagia following the stroke can significantly influence the extent of functional recovery in the chronic phase.^[1]^ As a result, conventional swallowing rehabilitation and 2-channel NMES often show limited effectiveness in improving swallowing function in chronic stroke patients. The mechanism of action for 2-channel NMES primarily involves a rehabilitative approach, namely, cumulative effects through muscle strengthening and facilitation of the swallowing reflex. It delivers electrical stimulation to the targeted swallowing muscles to enhance their function and coordination. Recent systematic reviews suggest the overall effectiveness of 2-channel NMES; however, optimization of channel number, electrode placement, and synchronization parameters remains a key challenge.^[5]^

In contrast, 4-channel NMES may function not only through rehabilitative mechanisms involving cumulative effects, but also by providing compensatory support for impaired muscle activation patterns during stimulation. Previous studies have shown that, under normal physiological conditions, the suprahyoid muscles are activated approximately 300 milliseconds prior to the infrahyoid muscles.^[15]^ However, conventional 2-channel NMES delivers simultaneous stimulation to both muscle groups, disregarding this natural activation sequence. This co-stimulation may result in hyolaryngeal depression, thereby diminishing the therapeutic benefits. In comparison, sequential stimulation of these muscles using 4-channel NMES is more physiologically aligned and may facilitate improved coordination of hyoid and laryngeal movements.^[10]^ Through these mechanisms, 4-channel NMES has been shown in previous studies to improve the maximal pressure and area of the velopharynx and mesopharynx, as well as the activation and nadir duration of the upper esophageal sphincter.^[9]^ These improvements may persist even during swallowing without electrical stimulation, possibly due to increased strength of the swallowing-related muscles.^[9]^

In this study, the 4-channel NMES was applied to improve swallowing function, but improvements were also observed in facial paresis and dysarthria. Previous study showed that NMES can induce sensory and motor cortical changes, resulting in plastic changes in the primary sensory cortex and in cortices associated with sensorimotor processing in individuals with chronic poststroke sequelae.^[10]^ Through these mechanisms, poststroke weakness such as facial palsy and dysarthria could have improved. In addition, due to cross-stimulation, current spread might have occurred, influencing tongue movement and thereby contributing to improvements in dysarthria.^[16]^

Because conventional swallowing rehabilitation was performed in parallel with 4-channel NMES, it is difficult to determine which intervention primarily contributed to the observed improvement. In addition, possible placebo effects or differences in rehabilitation adherence may have influenced the results. Moreover, as this study is based on a single-patient case report, the findings may have limited generalizability. These potential confounding factors should be considered when interpreting the findings. Further well-designed follow-up studies involving a larger patient population are needed to verify the specific effects of 4-channel NMES on dysphagia in chronic stroke patients.

The findings of this case report suggest that 4-channel NMES may be a potentially beneficial therapeutic option for improving swallowing function and quality of life in chronic stroke patients with dysphagia who do not respond to conventional 2-channel NMES. These observations are hypothesis-generatingand underscore the need for future large-scale randomized controlled trials comparing 4-channel NMES with 2-channel NMES and sham controls in patients with chronic stroke.

## Author contributions

**Conceptualization:** Jee Hyun Suh.

**Data curation:** Jee Hyun Suh, Jaewon Lee.

**Formal analysis:** Jee Hyun Suh.

**Funding acquisition:** Jee Hyun Suh.

**Investigation:** Jee Hyun Suh.

**Methodology:** Jee Hyun Suh, Jaewon Lee.

**Project administration:** Jee Hyun Suh.

**Supervision:** Jee Hyun Suh.

**Validation:** Jee Hyun Suh.

**Visualization:** Jee Hyun Suh.

**Writing** – **original draft:** Jee Hyun Suh.

**Writing** – **review & editing:** Jee Hyun Suh.
